# On Diabetes

**Published:** 1818-05

**Authors:** 


*For the London Medical and Physical Journal.*

### *On Diabetes*; by Dr. Trotter.

I HAVE much pleasure in announcing to the medical profession, through your pages, my successful treatment of two cases of Diabetes Mellitus, by *pure magnesia*. One of these was of long standing, in a man; the other in a female, of a shorter date. The prominent symptoms in both cases were —excessive thirst; profuse discharge of urine; voracious appetite; pain about the region of the stomach; emaciation of body; constipation, quick pulse, &c. The liquids daily used by the man, from ten to twelve quarts; and the urine made much about the same quantity, and sweet as sugar, gluey, and soon fermented. In the other case, the drink and urine about fourteen pints daily, of nearly the same quality, sweet flavour, cloggy, and let fall a dark-coloured sediment when it stood long, and grew frothy.

Having long observed the effect of Magn. Calcin. in complaints that seemed approaching to diabetes, I was at once induced to try it. I began with half a drachm, and a few grains of bark or columbo, gradually increasing it till the patient took upwards of a drachm and scruple thrice in twenty-four hours. The first day evinced a most wonderful change in the thirst, and in the quantity, taste, and smell, of the urine. When the full dose was given, excessive purging followed; but, instead of a weakening effect, the strength encreased, the voracious appetite declined to natural, the skin began to perspire freely, and the patient gained flesh.

In General Mulcaster's case, at Portsmouth, whom I attended for some time, as related by Dr. Rollo, I had seen the happy effects of a strict animal diet; but, in this man, I found it of no effect whatever. As soon as the magnesia was withdrawn, the urine became profuse and saccharine, and the desire for liquids returned. In this case, lime and the alkalies were all tried, without any salutary change; but recourse to magnesia again subdued the disease.

From motives of saving expense, a trial was made of Magn. Calcin. from the shop of a common druggist; but it was no better than prepared oyster-shells, and I was obliged to recur to the original medicines. I mention this, because the pure magnesia is not often prepared with all the attention it deserves, and is sometimes shamefully deteriorated by lime. Whoever, therefore, may be induced to imitate my practice in diabetes mellitus, must be cautious what magnesia they employ.

Having made this communication to your Journal, I shall reserve the cases, and the reflections which the novelty of the practice naturally suggest, to a work formerly announced, on the Diseases of the Labouring Class. As I obtain experience from a number of these people, who resort to me twice in the week for advice, from all parts of the country, my project daily assumes more importance; and my summary of cases puts on the appearance of a kind of *Medicina Statistica*, as a field not reaped before.

I ought to have mentioned, as a powerful testimony in favour of the pure magnesia, that I made no alterations of diet in the second case.

*Newcastle-on-Tyne; April* 4, 1818.

